# Similar survivorship at the 5-year follow-up comparing robotic-assisted and conventional lateral unicompartmental knee arthroplasty

**DOI:** 10.1007/s00167-022-07218-6

**Published:** 2022-11-14

**Authors:** Guido Maritan, Giorgio Franceschi, Roberto Nardacchione, Emanuele Furlan, Ilaria Mariani, Nicola Ursino, Riccardo D’Ambrosi

**Affiliations:** 1 Department of Knee Surgery, Policlinico Abano Terme, Abano Terme, Italy; 2 Institute for Maternal and Child Health IRCCS Burlo Garofolo, Trieste, Italy; 3 IRCCS Orthopedic Institute Galeazzi, Via Galeazzi 4, 20161 Milan, Italy; 4 Department of Biomedical Sciences for Health, University of Milan, 20133 Milan, Italy

**Keywords:** Knee replacement, Valgus, Knee osteoarthritis, Robotics, Osteoarthritis, Unicompartmental knee arthroplasty

## Abstract

**Purpose:**

This retrospective study aims to analyse the survivorship and functional outcomes of two samples with similar preoperative clinical and demographic data of lateral unicompartmental knee arthroplasty (UKA) performed with robotic and conventional surgery at a minimum 5-year follow-up.

**Methods:**

In this retrospective study, the clinical records of two cohorts for 95 lateral UKA implants were analysed. The first cohort consisted of 43 patients with cemented lateral UKA performed with the conventional procedure (Conventional group). The second cohort consisted of 52 patients who received robot-assisted cemented lateral UKA (Robotic group). Clinical evaluation of the two samples entailed evaluating the Knee Injury and Osteoarthritis Outcome Score divided into subscales (symptoms and stiffness, pain, function in daily living, function in sport and recreation and quality of life) for each patient. Revision was defined as the failure of the implant (periprosthetic joint infection, periprosthetic fracture or aseptic loosening), and survival was based on implant revision.

**Results:**

The mean follow-up time was 90.3 ± 9.1 months for the Conventional Group and 95.4 ± 11.0 months for the Robotic Group (n.s.). Each patient was clinically evaluated on the day before surgery (T_0_), at a minimum 1-year follow-up (T_1_) and at a minimum 5-year follow-up (T_2_). In both groups, all clinical scores improved between T_0_ and T_1_ and between T_0_ and T_2_ (*p* < 0.05); for both groups, no differences were noted in any clinical scores between T_1_ and T_2_ (n.s.). No significant differences in any clinical score were found between the two groups at each follow-up (n.s.). Survival analysis reported no differences between the two groups at the final 1-year follow-up, with three failures (2 aseptic loosening and 1 periprosthetic fracture) in the Conventional group and two failures (1 patellofemoral osteoarthritis and 1 inexplicable pain) in the Robotic group (n.s.).

**Conclusions:**

This study shows excellent clinical outcomes and revision rates in robotic arm-assisted and manual techniques for lateral UKA, with no clinical differences at medium- to long-term follow-up.

**Level of evidence:**

Level III—comparative study.

**Supplementary Information:**

The online version contains supplementary material available at 10.1007/s00167-022-07218-6.

## Introduction

Lateral unicompartmental knee arthroplasty (UKA) is still infrequently performed, despite possible therapeutic advantages. Although isolated lateral compartment arthritis affects 5% to 10% of people with knee osteoarthritis (OA), only 1% of knee arthroplasties involve this condition [1].

Robotic arm-assisted surgery has been introduced to help surgeons improve implant positioning. In particular, the robot can be useful in an uncommon procedure such as lateral UKA [2]. In the literature, several short-term studies report advantages of robotic surgery, such as improved accuracy of implantation, less soft-tissue damage, reduced postoperative pain and high patient satisfaction [3].

To date, few studies have compared robotic arm-assisted surgery and conventional surgery in the mid- or long-term, and no study has compared the lateral procedure yet [4].

The main purpose of this current retrospective study was to analyse the survivorship of two comparable samples of lateral UKA performed with robotic and conventional surgery at a minimum 5-year follow-up. The second aim was to compare clinical outcomes at different follow-ups.

It was hypothesised that in lateral UKAs, robotic arm-assisted surgery could lead to a higher rate of survivorship compared to the Conventional group with a lower rate of complications.

## Materials and methods

A retrospective analysis of the clinical records of 2 cohorts for a total of 95 lateral UKA implants was performed. All patients underwent surgery between 2011 and 2017. The first cohort consisted of 43 patients with cemented lateral UKA who underwent the standard nonrobotic procedure (conventional group). In comparison, the second cohort consisted of 52 patients who received robot-assisted cemented lateral UKA (Robotic group).

To eliminate selection bias, the inclusion and exclusion criteria for the study were restricted as follows for a more detailed comparison and to obtain similar preoperative values (Table 1): patients undergoing lateral UKAs procedure; minimum 60-month follow-up; age between 40 and 80 years; nonprevious surgery of the affected knee (except meniscectomy or anterior cruciate ligament [ACL] reconstruction); preoperative absence of systemic disease (e.g. diabetes, rheumatoid arthritis); preoperative BMI < 40.

**Table 1:** Demographics of the patient population

	Group		*p* value
	Conventional group *N* = 43 mean ± SD (%)	Robotic group *N* = 52 mean ± SD (%)	
Age	61.5 ± 8.5	60.9 ± 8.4	n.s
BMI	27.0 ± 2.8	26.2 ± 3.3	n.s
Sex, *n* (%)			
Female	37 (86.0)	41 (78.8)	n.s
Male	6 (14.0)	11 (21.2)	
Indications for surgery	19 primary OA (44.2) 16 OA post-meniscectomy (37.2) 5 OA post-ACL reconstruction (11.7) 1 OA post-tibial fracture (2.3) 2 AVN (4.6)	22 primary OA (42.4) 23 OA post-meniscectomy (44.2) 3 OA post-ACL reconstruction (5.8) 2 OA post-tibial fracture (3.8) 2 AVN (3.8)	
Side, *n* (%)			
Left	13 (30.2)	14 (26.9)	n.s
Right	30 (69.8)	38 (73.1)	
T_1_ (months)	13.0 ± 1.1	13.5 ± 1.4	n.s
T_2_ (months)	90.33 ± 9.1	95.38 ± 11.0	n.s

*BMI* body mass index, *OA* osteoarthritis, *ACL* anterior cruciate ligament, *AVN* avascular necrosis

*n.s.* Not significant

All the procedures were performed in two different high-volume centres specialised in knee surgery:

1. IRCCS Istituto Ortopedico Galeazzi, Milan, Italy. All UKAs in the Conventional Group were performed by a single surgeon (43 patients).
2. Policlinico di Abano Terme, Abano Terme, Italy. All robotic group procedures were performed by four different surgeons (52 patients).

The indications for surgery were as follows: Kellgren–Lawrence of the lateral compartment grade III or IV OA; avascular necrosis or osteonecrosis isolated of the lateral femoral condyle; idiopathic or secondary osteoarthritis of the lateral femoral compartment of the knee; knee flexion > 100°; flexion contracture < 15°; valgus deformity (measured on hip–knee–ankle angle) < 10°; integrity of cruciate and collateral ligaments; osteoarthritis of the medial compartment and patellofemoral grade I or II according to Kellgren–Lawrence classification [5].

Magnetic resonance imaging (MRI) was assessed preoperatively to confirm the anatomical integrity of the cruciate and collateral ligaments in all patients and the absence of OA of the medial and patellofemoral knee compartment.

### Patient recruitment

**Fig. 1:**
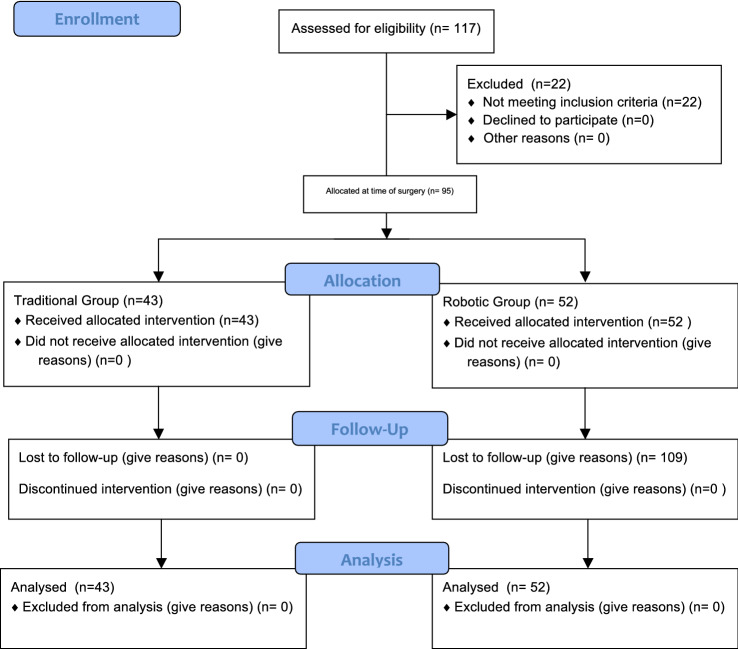
CONSORT flowchart

A total of 117 patients were initially screened. Of them, 22 were deemed ineligible for the following reasons: severe OA of the patella requiring resurfacing (*N* = 9), previous high tibial osteotomy (*N* = 8) and previous femoral/tibial fracture (*N* = 5). A total of 95 patients were included in the present study: 52 were allocated to the Conventional group and 43 to the Robotic group (Fig. 1) [6]. No patients were lost to follow-up.

### Surgical technique: conventional group

All patients were placed in a supine position on a standard operating table after spinal anaesthesia had been induced, as *the knee and ipsilateral hip should be freely mobile without the use of a tourniquet.* Good exposure was obtained with a skin incision, starting from the lateral margin of the patella to a point approximately 4–5 cm distal to the joint line. Deep exposure was achieved with a lateral parapatellar approach through the subcutaneous tissues to the joint capsule, and the patella was medially dislocated. Inspection of the patellofemoral and medial compartments was routinely performed. The ACL status was checked. Lateral and intercondylar osteophytes were removed, and an anterior tibial precut was performed to gain adequate posterior and articular view and access. For balancing, a spacer block was inserted into the joint space until the anterior stop contacted the anterior tibia to assess the gap. The spacer block was placed to sit flat on the resected tibial surface to ensure that the proper amount of distal femoral bone was resected.

In all cases, the fixed-bearing ZUK Unicompartmental Knee (LimaCorporate, Villanova di San Daniele del Friuli, Udine, Italy) was implanted using the corresponding instrumentation, extramedullary tibial guide and femoral and tibial cutting guides. All components were cemented using Refobacin^®^ Bone Cement R (Zimmer Biomet, Warsaw, Indiana, USA). Tibial coverage was maximised without any overhang while targeting the natural tibial slope [7].

### Surgical technique: robotic group

All the prostheses were fixed-bearing metal-backed UKAs (Restoris MCK, Stryker, Mahwah, New Jersey) implanted with a semiautonomous robot, the MAKO robotic arm system (Stryker). A lateral parapatellar approach was performed [8].

Before the surgery, a 3D model of the patient’s knee was created from a preoperative CT scan. Then, surgical planning was performed, defining the size and position of the tibial and femoral components, the amount of bone resection and the implant alignment. The planning was changed after bone registration and pose capture, applying varus stress to correct the valgus deformity and tension in the lateral collateral ligament. Once gap balancing and implant tracking were checked, bone resection was performed using a 6 mm burr. The final implants were cemented using Biomet^®^ Bone Cement R (Zimmer Biomet, Warsaw, Indiana, USA).

### Rehabilitation protocol

Both patient groups followed the same rehabilitation protocol, which involved passive mobilisation on the same day of surgery; from day one, patients were started on active progressive joint mobilisation and assisted walking with two crutches. Gradually and according to each patient, recommendations were made to increase the load during walking and continue with isometric muscle toning exercises until patients could walk independently without the use of walking aids [9].

### Survivorship

A revision was defined as the failure of the implant (periprosthetic joint infection [PJI], periprosthetic fracture, aseptic loosening or revision with TKA for medial or patellar cartilage deterioration), and survival was based on implant revision. PJI was diagnosed according to the New Definition for Periprosthetic Joint Infection: From the Workgroup of the Musculoskeletal Infection Society [10]. Periprosthetic fractures were defined as fractures of the femur or tibia occurring within 15 cm of the joint line or 5 cm of the endomedullary stem if present [11]. Patients were classified as having aseptic loosening if they had symptoms including pain, instability or swelling and radiographic evidence of loosening and did not meet the Musculoskeletal Infection Society criteria for infection [12].

### Clinical evaluation

All clinical assessments were performed by two independent clinicians who were not involved in the index surgery. The clinical evaluation entailed evaluating the Knee Injury and Osteoarthritis Outcome Score (KOOS), which is divided into subscales (symptoms and stiffness, pain, function in daily living, function in sport and recreation and quality of life) for each patient. Each patient was clinically evaluated on the day before surgery (T_0_), at a minimum 1-year follow-up (T_1_) and at a minimum 5-year follow-up (T_2_) [13].

All the procedures involving human participants in this study followed the institutional or national research committee ethical standards, the 1964 Helsinki Declaration and its later amendments or comparable ethical standards. The study followed the Strengthening the Reporting of Observational Studies in Epidemiology (STROBE) guidelines. Informed consent was obtained from all the participants included in the study [14]. Appropriate ethical approval was also obtained from the local ethics committee (Ethical Committee of San Raffaele Hospital—CE 236/2017).

### Statistical analysis

Summary statistics are presented as the mean and standard deviation (SD) values or absolute frequencies and percentages. After testing the distribution of continuous variables, a t test or a Wilcoxon–Mann–Whitney test was performed to assess preoperative differences between the Conventional and Robotic groups, and a chi-square test was used to evaluate the categorical variables*.*

The groups’ KOOS total and subscores were compared at each time point with a t test or a Wilcoxon–Mann–Whitney test as appropriate. Then, for each group, pre- and postoperative scores were compared with a paired t test or a Wilcoxon signed-rank test.

Failures were recorded for each group, and Fisher’s exact test was performed to assess any differences in the total number of failures between the conventional and robotic groups. In addition, survival curves were estimated for each group to account for the time of failure onset from surgery. A Cox regression model was created using ‘failure’ as an independent variable and the specific group as a covariate. Age, BMI, sex and surgical side were also added as covariates to the Cox model, but no statistical significance was found. All tests were two-sided, and a *p* value of less than 0.05 was considered statistically significant. Statistical analyses were conducted in R (version 4.1.1).

### Sample size

An estimated sample of 80 subjects, 40 for each group, was required to compare KOOS scores between groups with a two-sided Wilcoxon–Mann–Whitney test, assuming a mean difference of 20, an SD of 20 for both groups, a 5% alpha and 99% power. Given the same parameters, this sample also had 99% power to detect a prepost difference using a Wilcoxon signed-rank test [15].

## Results

No preoperative differences were noted between the two groups (n.s.). Detailed results are reported in Table 1.

### Survivorship

**Fig. 2:**
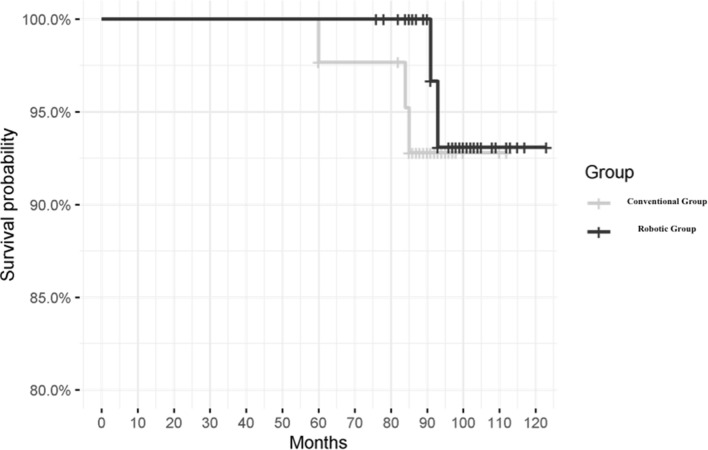
Kaplan–Meier curves and survival probability for each group

Survival analysis reported no differences between the two groups at the final 5-year follow-up, with three failures in the Conventional group and two in the Robotic group (*p* = 0.4). Details are reported in Table 2. Figure 2 shows the Kaplan–Meier curves for survivorship in both groups.

**Table 2:** Clinical comparison between groups at each follow-up

	Traditional *N* = 43**	Robot *N* = 52**	Group comparison (traditional vs robot)	Time comparison (T_0_ vs T_1_) *p* value	
	Mean ± SD	Mean ± SD	*p* value	Traditional	Robot
KOOS total score					
T_0_	36.4 ± 7.6	37.3 ± 7.3	0.6	< 0.001*	< 0.001*
T_1_	85.6 ± 10.7	87.7 ± 10.7	0.3		
Symptoms and stiffness					
T_0_	52.0 ± 13.4	52.6 ± 13.2	0.8	< 0.001*	< 0.001*
T_1_	91.8 ± 14.8	95.0 ± 10.5	0.5		
Pain					
T_0_	38.7 ± 9.6	38.9 ± 9.4	0.9	< 0.001*	< 0.001*
T_1_	93.7 ± 8.5	95.0 ± 8.0	0.5		
Function, daily living					
T_0_	39.2 ± 9.3	38.9 ± 9.0	0.9	< 0.001*	< 0.001*
T_1_	95.1 ± 8.2	95.8 ± 8.1	0.6		
Function, sports					
T_0_	12.0 ± 9.5	14.8 ± 10.2	0.2	< 0.001*	< 0.001*
T_1_	59.1 ± 18.1	61.2 ± 25.0	0.7		
Quality of life					
T_0_	26.6 ± 8.7	28.1 ± 8.4	0.4	< 0.001*	< 0.001*
T_1_	88.7 ± 15.0	91.00 ± 15.3	0.2		

*KOOS* knee injury and osteoarthritis outcome score

T_0_ = Pre-operative follow-up

T_1_ = Final follow-up

*Statistical significant value (*p* < 0.05)

**Scores at T_1_ were evaluated on 40 and 50 patients for traditional and robot group, respectively, because of failures.

The Cox model showed no difference in survivorship between the two groups, even when corrected for BMI, age, sex and side (n.s.).

### Clinical outcomes

In both groups, all clinical scores improved between T_0_ and T_1_ and between T_0_ and T_2_ (p < 0.05); for both groups, no differences were noted in any clinical scores between T_1_ and T_2_ (n.s.)

**Fig. 3:**
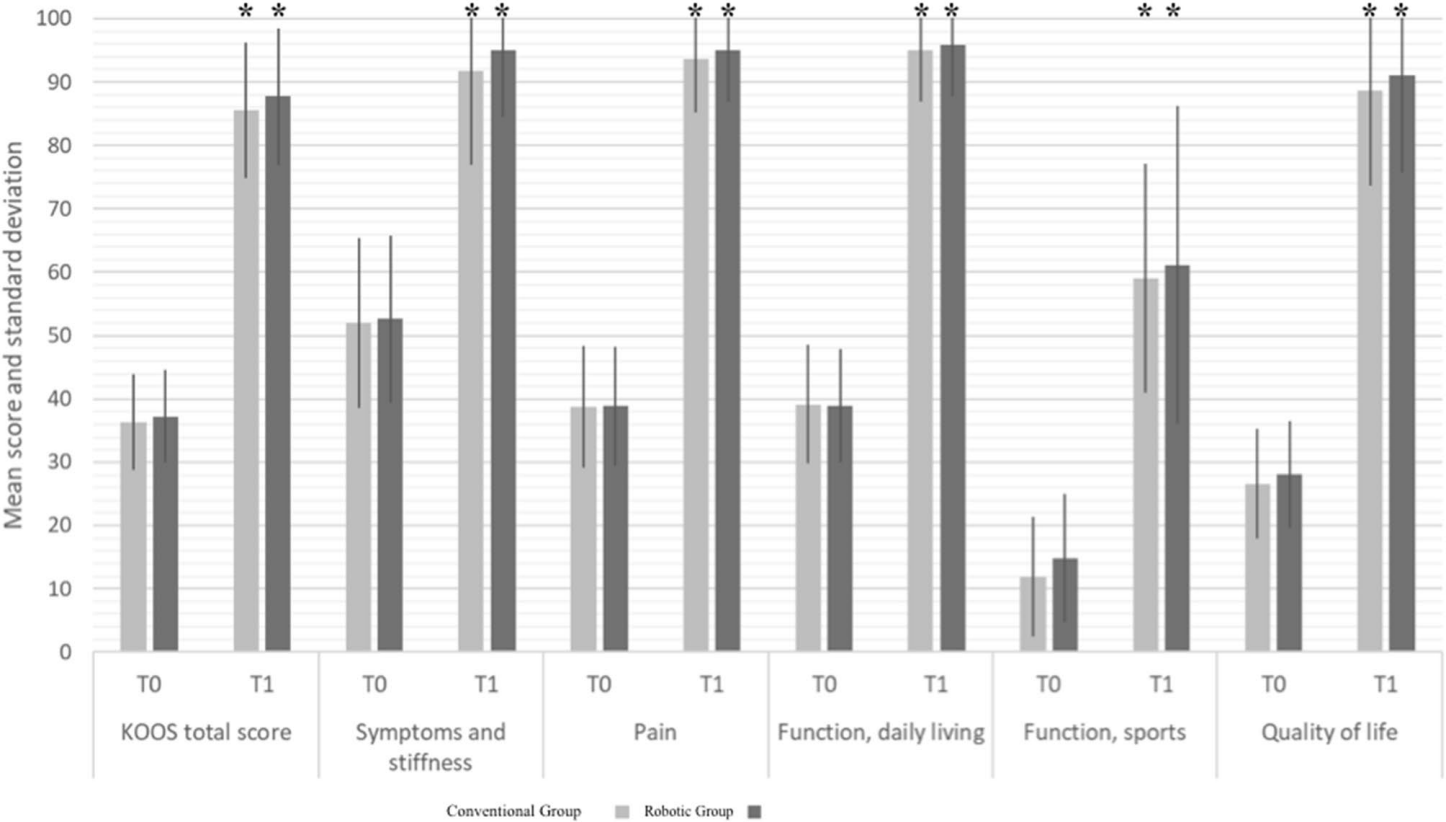
Clinical scores for both groups at follow-up. *Statistically significant improvement (*p* < 0.05) versus T_0_. T_0_ = preoperative follow-up; T_1_ = final follow-up

No significant differences in any clinical score were found between the two groups at each follow-up (n.s.). Detailed results are reported in Table 3and Fig. 3.

**Table 3:** Analysis of failures and complications for both groups during the study period

Month	Number of patients at risk	Number of failures	Survival probability % (95% CI)
Traditional group			
60	43	1 (aseptic loosening)	97.7 (93.3–100.0)
84	42	1 (aseptic loosening)	95.2 (89.0–100.0)
85	41	1 (periprosthetic fracture)	92.8 (85.3–100.0)
Robot group			
91	52	1 (patellofemoral OA)	96.7 (90.5–100.0)
93	51	1 (inexplicable pain)	93.1 (84.3–100.0)

*OA* osteoarthritis

Difference in survival time: Chi-squared test *p* value = 0.4

## Discussion

The most important finding of the present study was that both techniques showed excellent results, with a satisfactory mean postoperative KOOS score and a significant improvement between the pre- and postoperative mean scores on every subscale. The mean KOOS score at a minimum of 5 years was 85.6 for the Conventional group and 87.7 for the Robotic group, with preoperative means of 36.4 and 37.3, respectively. Although the functional score was slightly higher in the robotic group, especially in the symptoms, stiffness and quality of life categories, this difference was not statistically significant. However, a nonsignificantly lower rate of complications was found, particularly for aseptic loosening, in the robotic group (2 versus 0).

The mean KOOS score and the significant difference between the pre- and postoperative scores show how UKA is efficient not only in pain relief but also in the improvement of quality of life. Our results are similar to those reported in the literature after both manual and robotic-assisted procedures [1]. While the clinical benefits are well established worldwide, the advantages in terms of the survivorship of partial knee arthroplasty are put into question. In fact, the current literature reports a higher revision rate of UKA compared to TKA [16].

In this study, the overall survivorship was approximately 93%, and our results seem to be comparable to those of Mergenthaler et al., who reported survivorship rates of 89% and 96% for medial and lateral UKAs with manual and robotic surgery, respectively, at a 2-year follow-up [17].

In short-term follow-up, Thein and Zambianchi reported a 100% survivorship rate in lateral UKAs [2, 18].

In the mid-term, at 6 years, a recent systematic review of 38 studies (seven evaluating the lateral procedure) reported a survivorship rate of 96% considering both medial and lateral UKAs [19].

In the analysis of the survivorship of unicompartmental prostheses performed manually, our results are comparable to those of other cohort studies and the National Joint Registry for England, Wales, Northern Ireland and the Isle of Man, with a survivorship of 93% in 2,052 partial lateral arthroplasties at 5 years [20, 21]. Registry studies describe an increasing rate of revisions every 10 years [20, 21].

Current literature confirms that the leading cause of revision is the progression of osteoarthritis in other compartments of the knee, responsible for 24–43% of implant failures and aseptic loosening, particularly of the tibial component, which accounts for 16–28% of the revisions [20–22]. The current study reported no aseptic loosening in the robotic group, confirming that robotic-assisted surgery is associated with a lower risk of aseptic loosening [22]. A recent cadaveric study observed better precision in the position of the tibial and femoral components with a semiactive robot compared to manual surgery [23]. A similar finding was reported by Bell et al., who analysed 62 UKAs made with the MAKO robot and 58 Oxford implants made manually through a CT scan, concluding that the technology ensures better positioning of the components in the coronal, sagittal and axial planes [24].

It is known that among the causes of failure is the surgeons’ yearly volume of UKAs. Moreover, lateral UKA could be technically demanding, with multiple difficulties of approach, component positioning, limb alignment and balancing [25].

Particularly, in lateral UKA, the positioning of the tibia plays a key role; a recent study showed how excessive external rotation of the tibial component could negatively influence postoperative outcomes [26, 27]. Robotic-assisted UKA shows a better rate of joint line restoration than conventional UKA [3].

Regarding robotic procedures, Kayani et al. claim that robotic TKA does not have a learning curve effect for achieving the planned implant positioning. It is unclear whether our results would be the same for low-volume UKA surgeons or those with more experience with the technology [3].

These findings inform shared decision-making and can help surgeons decide on ideal implants and techniques. In particular, no evidence has been demonstrated regarding the superiority of robotic arm-assisted procedures. Furthermore, it is not an economical procedure and will only be cost-effective compared with conventional UKA when the annual case volume exceeds 94 cases per year. It is not cost-effective at low-volume or medium-volume arthroplasty centres [28].

Several limitations of the present study must be considered.

The first limitation is its retrospective design: only the patients who could be contacted and were able to be visited were included. Hence, a bias in the selection of the patients may be present.

Second, all surgeries were carried out by high-volume UKA surgeons in a high-volume unit with more than 200 UKAs per year [29], so our findings may not be generalisable to institutions where UKAs are not performed as frequently.

Then, pre- and postoperative radiographs were not analysed. The lack of radiographs is because the study is a retrospective study and the patients had radiographs at centres other than those where the surgery was performed, or the radiographs were not taken according to the protocol.

Another limitation is the use of different lateral UKAs, but the current literature confirms that both implants are safe, leading to satisfactory clinical and radiographic results [4]. Retrospective studies, however, have several limitations owing to their designs. Since they depend on the review of charts that were originally not designed to collect data for research, some information is bound to be missing [30]. These limitations negatively impacted the quality of our conclusions, increasing the risk of selection, detection and performance biases.

This study is only a retrospective study with the well-known risks of selection bias, which was reduced with restricted inclusion and exclusion criteria. A prospective study that excludes confounding factors by good experimental design should be conducted, even a randomised controlled trial.

Finally, the conventional and robot-assisted procedures were performed in two different hospitals. However, a similar pain control therapy and rehabilitation protocol were followed to reduce possible bias.

## Conclusion

This study shows excellent clinical outcomes and revision rates in robotic arm-assisted and conventional techniques for lateral UKA, with no clinical differences at medium- to long-term follow-up.

## Supplementary Information

Below is the link to the electronic supplementary material.

Supplementary file1 (XLSX 46 KB)

## Data Availability

Raw data have been submitted as supplementary material to the journal.
